# Whole-genome sequencing and annotation of probiotic *Bacillus amyloliquefaciens* COFCAU_P1

**DOI:** 10.1128/mra.01483-25

**Published:** 2026-05-14

**Authors:** Tanmoy Gon Choudhury, Md Idrish Raja Khan, Partha Sarathi Tripathy, Suraj Kumar, Ratan Kumar Saha, Dibyendu Kamilya

**Affiliations:** 1 Department of Aquatic Health and Environment, College of Fisheries, Central Agricultural University (Imphal), Lembucherra, Tripura, India; 2 Department of Aquatic Animal Health Management, College of Fisheries Science, Chaudhary Charan Singh Haryana Agricultural University, Hisar, Haryana, India; 3 College of Fisheries, Rani Lakshmi Bai Central Agricultural University, Jhansi, India; 4 Techno India University, Tripura 740975 , IndiaAgartala, Tripura; 5 Agricultural and Food Engineering Department, Indian Institute of Technology Kharagpur 30133, Kharagpur, West Bengal, India; University of Manitob, Winnipeg, Manitoba, Canada

**Keywords:** probiotics

## Abstract

*Bacillus amyloliquefaciens* COFCAU_P1, an autochthonous probiotic from *Labeo rohita*, was genome sequenced (Illumina NovaSeq; GenBank JBHEQH000000000). Its 4.09 Mb genome encodes 4,351 genes linked to stress tolerance, nutrient utilization, antimicrobial defense, enzymes, and immunomodulation. Phylogenomics and functional evidence confirm its safety and probiotic potential for aquaculture applications in aquaculture systems.

## ANNOUNCEMENT

*Bacillus amyloliquefaciens* COFCAU_P1 is an autochthonous probiotic strain isolated from the intestine of *Labeo rohita* (Rohu) (1). The strain displays strong antagonistic activity against major fish pathogens, tolerance to a wide pH range (2–9), and high bile concentrations (up to 10%) and exhibits high mucus adhesion, hydrophobicity, and aggregation abilities. It is non-hemolytic and produces proteinase, amylase, lipase, and cellulase. Genetic screening confirmed multiple probiotic-associated marker genes, and feeding trials in *L. rohita* demonstrated enhanced immune responses and resistance to *Aeromonas hydrophila* (2). These combined attributes support COFCAU_P1 as a potent and safe probiotic candidate for aquaculture (3).

**Fig 1:**
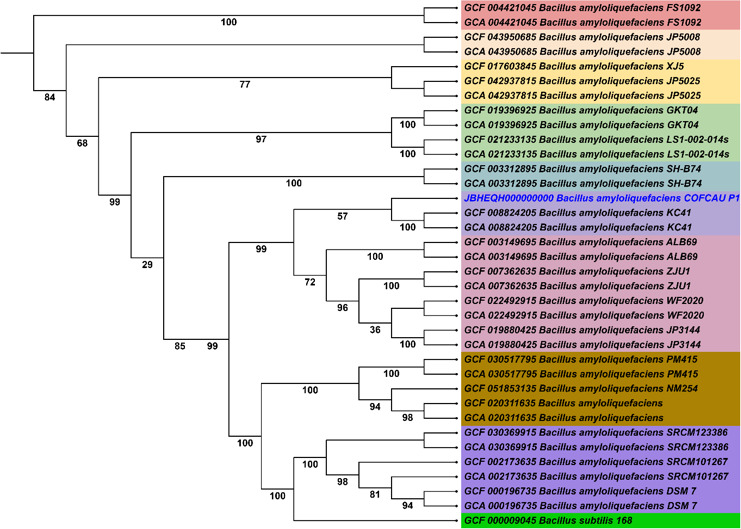
Phylogenomic tree contains the isolated *B. amyloliquefaciens* COFCAU_P1 from this fish and other strains from NCBI.

A single purified colony was transported to the sequencing laboratory in tryptone soy agar plate. The bacteria was grown in tryptone soy broth at 37°C under agitation. Genomic DNA was extracted using the phenol–chloroform method and quality-checked using a NanoDrop spectrophotometer (4). Whole-genome sequencing was performed on the Illumina NovaSeq 6000 platform using 2 × 150 bp paired-end reads. Raw reads were quality-checked and trimmed using FastQC v0.12.0 and Trimmomatic v0.40 before downstream analysis. High-quality sequencing reads were assembled using SPAdes v4.2.0, and the assembled genome was annotated using the National Center for Biotechnology Information (NCBI) Prokaryotic Genome Annotation Pipeline (PGAP, v6.8). Taxonomic assignment of the assembled genome was performed using GTDB-Tk v2.6.1. Assembly quality assessment using QUAST v5.3.0 showed a high-quality 4.09 Mb genome with 46.94% guanine-cytosine (GC) content and 109× coverage. BUSCO v6.0.0 and CheckM v1.2.4 estimated ~99% completeness with <1% contamination, indicating a complete, low-contamination assembly. Functional categorization of probiotic-associated genes was carried out using the Bacterial and Viral Bioinformatics Resource Center (BV-BRC) (5). Whole-genome phylogenetic analysis was performed with GToTree (v1.8.13) (6), and the resulting phylogeny was visualized using iTOL (v6) (7) (Fig. 1). The assembled genome is 4,090,354 bp and contains 4,351 genes, including 4,118 protein-coding sequences. Eighty-five RNA genes were identified (70 tRNAs, 5 ncRNAs, and multiple copies of 5S, 16S, and 23S rRNAs). A total of 148 pseudogenes were predicted (Table 1).

**TABLE 1:** Genomic and probiotic functional features of *Bacillus amyloliquefaciens* COFCAU_P1

Feature category	Description
Sequencing technology	Illumina NextSeq
Assembly method	NCBI PGAP (best-placed reference protein set + GeneMarkS-2+)
Genome coverage	High-depth Illumina coverage
Genome length (bp)	4,090,354
GC content (%)	46.940,674
Total genes	4,351
Protein-coding genes	4,118
RNA genes	85
tRNA genes	70
rRNA genes	5S × 3, 16S × 4, 23S × 3
ncRNAs	5
Pseudogenes	148
BioProject accession	PRJNA1158341
BioSample accession	SAMN43535920

## Data Availability

The WGS sequence of *Bacillus amyloliquefaciens* COFCAU_P1 can be accessed from NCBI GenBank JBHEQH000000000. The project is deposited under BioProject PRJNA1158341 and BioSample SAMN43535920. The raw sequence data are available in the Sequence Read Archive (SRA) database under accession number SRR36553005.
